# Multi-color RGB marking enables clonality assessment of liver tumors in a murine xenograft model

**DOI:** 10.18632/oncotarget.23312

**Published:** 2017-12-14

**Authors:** Michael Thomaschewski, Kristoffer Riecken, Ludmilla Unrau, Tassilo Volz, Kerstin Cornils, Harald Ittrich, Denise Heim, Henning Wege, Ercan Akgün, Marc Lütgehetmann, Jan Dieckhoff, Michael Köpke, Maura Dandri, Daniel Benten, Boris Fehse

**Affiliations:** ^1^ Research Department of Cell and Gene Therapy, Department of Stem Cell Transplantation, University Medical Center (UMC) Hamburg-Eppendorf, Hamburg, Germany; ^2^ Department of Medicine, Gastroenterology and Hepatology, UMC Hamburg-Eppendorf, Hamburg, Germany; ^3^ Diagnostic and Interventional Radiology, UMC Hamburg-Eppendorf, Hamburg, Germany; ^4^ Department of Gastroenterology, Helios Klinikum Duisburg, Duisburg, Germany

**Keywords:** HCC, insertional mutagenesis, RGB marking, LeGO vectors, hTERT

## Abstract

We recently introduced red-green-blue (RGB) marking for clonal cell tracking based on individual color-coding. Here, we applied RGB marking to study clonal development of liver tumors. Immortalized, non-tumorigenic human fetal hepatocytes expressing the human telomerase reverse transcriptase (FH-hTERT) were RGB-marked by simultaneous transduction with lentiviral vectors encoding mCherry, Venus, and Cerulean. Multi-color fluorescence microscopy was used to analyze growth characteristics of RGB-marked FH-hTERT *in vitro* and *in vivo* after transplantation into livers of immunodeficient mice with endogenous liver damage (uPA/SCID). After initially polyclonal engraftment we observed oligoclonal regenerative nodules derived from transplanted RGB-marked FH-hTERT. Some mice developed monochromatic invasive liver tumors; their clonal origin was confirmed both on the molecular level, based on specific lentiviral-vector insertion sites, and by serial transplantation of one tumor. Vector insertions in proximity to the proto-oncogene MCF2 and the transcription factor MITF resulted in strong upregulation of mRNA expression in the respective tumors. Notably, upregulated MCF2 and MITF expression was also observed in 21% and 33% of 24 human hepatocellular carcinomas analyzed. In conclusion, liver repopulation with RGB-marked FH-hTERT is a useful tool to study clonal progression of liver tumors caused by insertional mutagenesis *in vivo* and will help identifying genes involved in liver cancer.

## INTRODUCTION

As the incidence of advanced stage hepatocellular carcinoma (HCC) is increasing and affected patients face a poor prognosis despite new medical treatment options, there is a high need for alternative treatment strategies. To this end, more comprehensive understanding of the molecular pathogenesis of HCC is required. In addition, preclinical research on targeted HCC therapies has been hampered by the absence of slow-growing humanized *in-vivo* models.

Although many risk factors, such as hepatitis B virus (HBV) infection, are well known, processes of molecular HCC evolution and its malignant progression remain to be elucidated. This includes a potential role of virus-insertion mediated oncogene dysregulation. HBV, which is a pararetrovirus, replicates via reverse transcription with stable integration of subgenomic fragments in up to 90% of HCC [1]. These integrations are unregulated and randomly distributed throughout the hepatocyte genome. Several studies have suggested a role of these HBV insertions in HCC development, although its actual contribution has not been completely proven [2, 3].

In contrast, the causal role of virus-insertion mediated oncogene dysregulation has long been established for the development of other cancers and hematologic malignancies. This had become the basis for the use of insertional mutagenesis (IM) to identify cancer-related genes using replication-competent retroviruses [4, 5]. Subsequently it has been shown that retroviral vectors can also be used for IM, e.g. to identify (protoonco-) genes involved in leukemia and cancer [6–9]. More recently, IM was successfully transferred to liver carcinogenesis using lentiviral vectors in newborn mice with different liver damage models [10], which led to identification of previously unknown liver cancer-associated genes. In an alternative approach, we applied retroviral IM to transform human fetal hepatocytes expressing the human telomerase reverse transcriptase (FH-hTERT) *in vitro* and identified RIPK4 as a tumor suppressor gene [11]. However, in that study the IM approach was not applied *in vivo*, since FH-hTERT did not sufficiently engraft and proliferate after subcutaneous transplantation in nude mice.

We have previously developed a lentiviral vector platform referred to as LeGO vectors for permanent cell marking, transgene overexpression and gene suppression by RNA interference [12, 13]. This vector system was used to introduce red-green-blue (RGB) marking, a novel labeling technique to investigate normal and aberrant organ regeneration on a clonal level *in vivo* [14–17].

In order to elucidate the applicability of RGB marking to study engraftment and proliferation of FH-hTERT in a liver regeneration model, we here used orthotopic transplantation of RGB-marked FH-hTERT into the endogenously damaged livers of uPA/SCID mice. Based on the positive results, we extended this model to combine RGB marking with IM. Jointly applied, the two techniques allowed us to investigate development of FH-hTERT-derived human liver tumors *in vivo* and enabled identification of proto-oncogenes potentially involved in HCC oncogenesis.

## RESULTS

### Cell characteristics and RGB transduction

**Figure 1:**
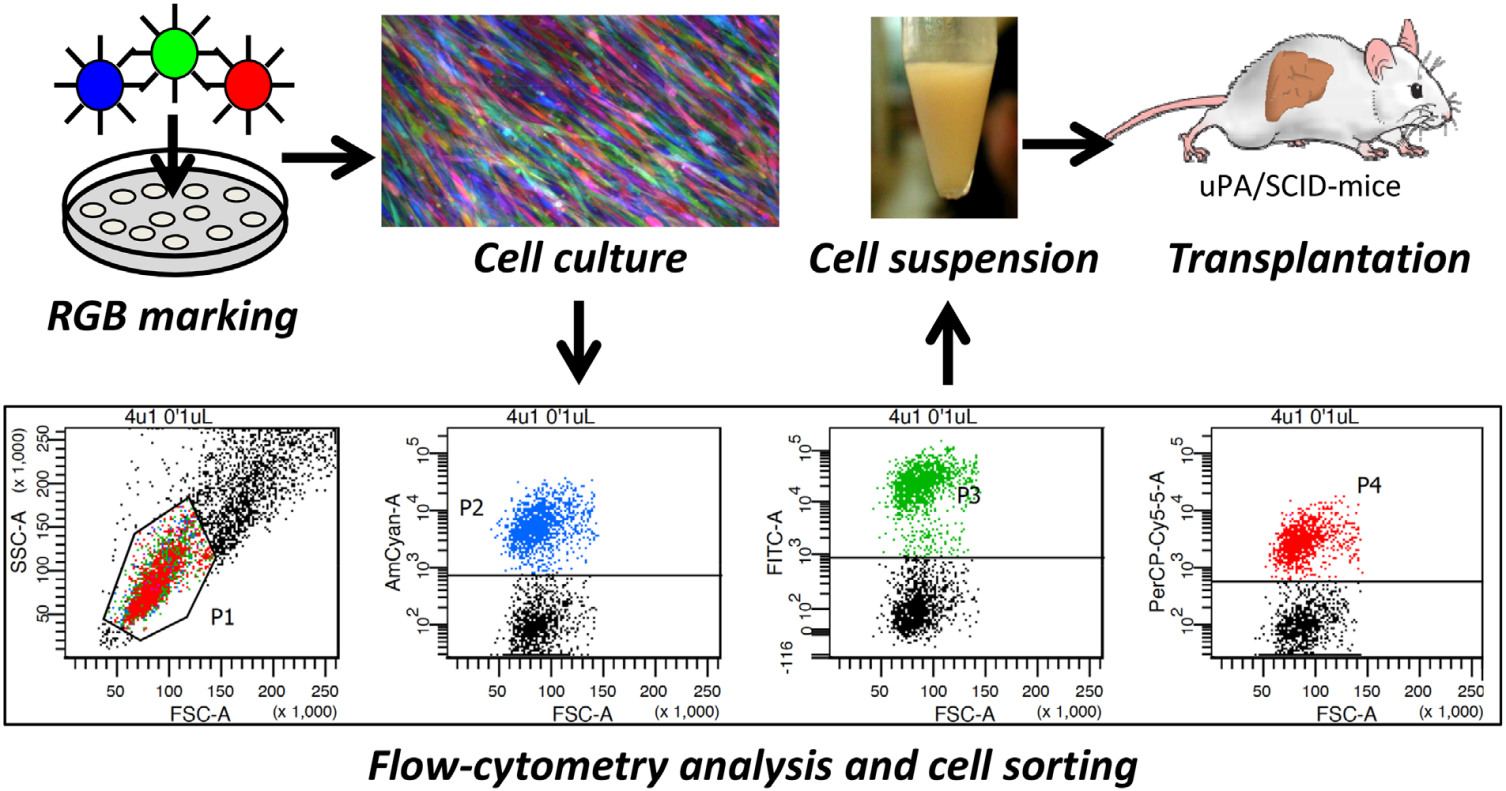
RGB marking and transplantation of FH-hTERT FH-hTERT were labeled according to the RGB-marking principle that facilitates clonal cell labelling of transduced cells with a variety of different colors. Untransduced FH-hTERT were removed by FACS, and remaining RGB-marked FH-hTERT were transplanted into hemizygous uPA/SCID-mice.

We followed the RGB principle to mark FH-hTERT cells. Efficient transduction with all three LeGO vectors as shown by flow cytometry resulted in a large variety of different colors (Figure 1). Moreover, fluorescence microscopy revealed a highly motile growth pattern of FH-hTERT (Figure 1). Thus, in contrast to other cells [14] single FH-hTERT clones can hardly be identified *in vitro* based on RGB marking.

### Cell engraftment and cell proliferation

FH-hTERT were previously shown to engraft in the liver of recipient mice, where they continue to express hepatocyte-specific proteins. However, there was only limited evidence for proliferative activity of engrafted FH-hTERT *in vivo* [18]. In order to improve proliferation, we made use of the well-established uPA/SCID mouse transplantation model [19–21], in which the liver-toxic uPA transgene induces subacute liver failure in newborn animals, thereby mediating a growth advantage for transplanted hepatocytes. Since this effect alone was not sufficient for significant repopulation of livers transplanted with FH-hTERT in our pilot experiments (data not shown), we additionally used the alkaloid monocrotaline (MCT) to improve cell engraftment by permeabilizing the sinusoidal endothelium [22, 23].

**Figure 2:**
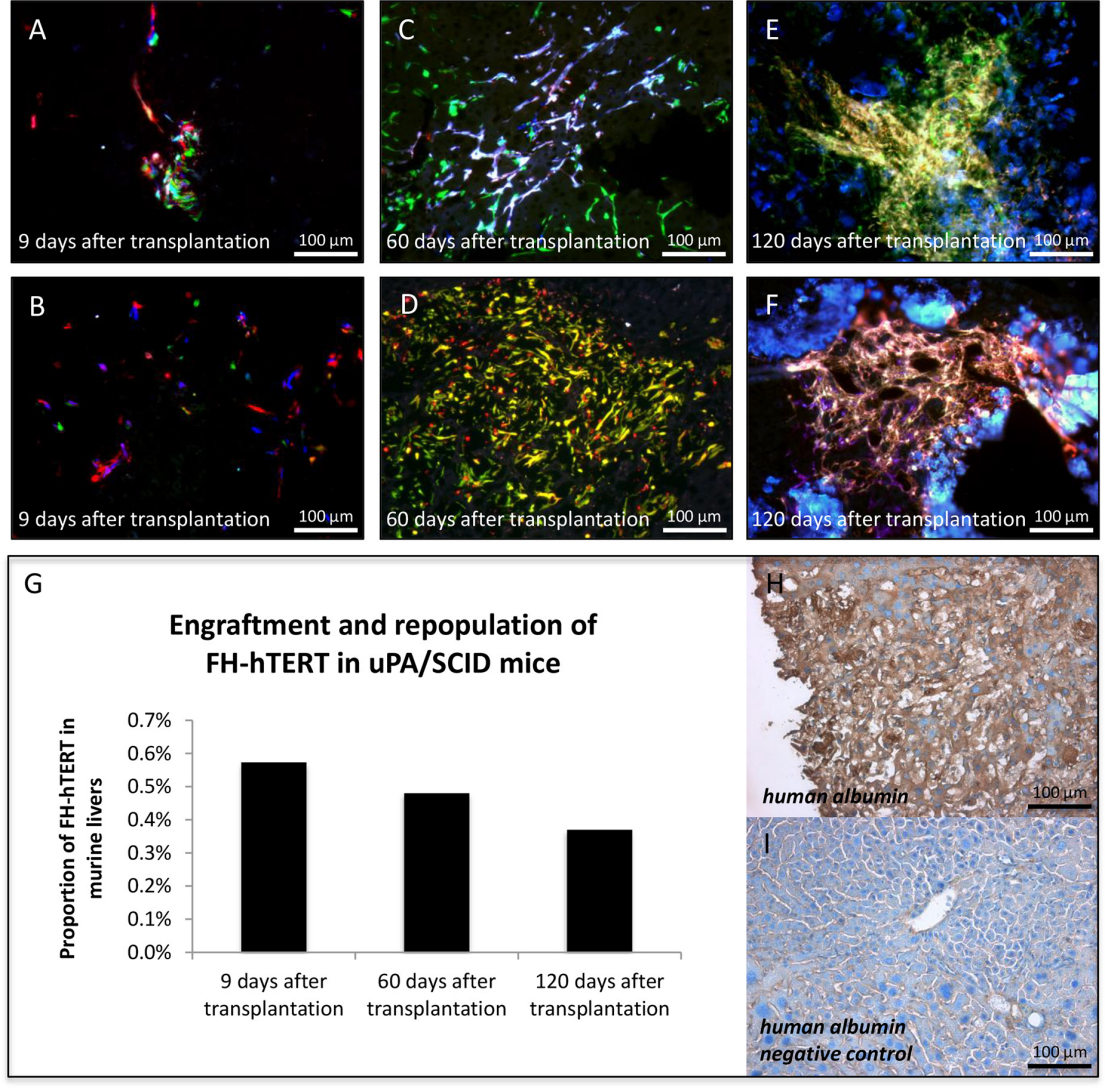
Clonal growth of FH-hTERT in mouse livers Transplanted uPA/SCID-mice were sacrificed at different time points and RGB marking allowed visualization of clonal FH-hTERT growth in the liver. **(A-B)** Nine days after transplantation multiple colors in cell clusters indicated polyclonal engraftment. **(C-D)** After 60 days, observed clusters were larger while numbers of colors in cell clusters decreased to two or three, indicating oligoclonal growth of some engrafted cells. **(E-F)** After 120 days, cell densities in repopulated areas were increased as compared to day 60, whereas cluster sizes and color numbers were in the same range. **(G)** The relative content of human DNA in mouse livers slightly decreased over time, indicating that only a proportion of the engrafted cells proliferated in transplanted mice. **(H-I)** Transplanted FH-hTERT retained human albumin production, while unrepopulated areas of the mouse liver were negative for human albumin.

1×10^6^ FH-hTERT per mouse were transplanted into 10 hemizygous uPA/SCID mice by intrasplenic injection. To assess the kinetics of engraftment, a time-course analysis of cell proliferation was performed. Early FH-hTERT engraftment was analyzed in one mouse sacrificed 9 days after transplantation. Fluorescence microscopy of liver cryosections showed transplanted cells mainly in periportal areas of most liver lobes. Several spots of multiple RGB-marked FH-hTERT with mixed fluorescence colors indicated clustered, polyclonal engraftment of transplanted cells (Figure 2A–2B). In two mice sacrificed 60 days after transplantation, some of the engrafted FH-hTERT had proliferated resulting in larger regenerative nodules of up to 1 mm size. RGB marking revealed that these nodules were now oligoclonal as reflected by the presence of only few, mostly 2-3 different colors within outgrown cell nodules (Figure 2C–2D). 120 days after cell transplantation, the size of transplanted cell nodules in two further mice had not significantly increased, compared to the 60-day time point, but cell density was higher at 120 days, and most nodules were now mono- or oligoclonal, i.e. they displayed one or two fluorescence colors, only (Figure 2E–2F).

Since this was the first systematic analysis of FH-hTERT proliferation in an orthotopic xenograft mouse model, liver repopulation was measured by quantitative real-time PCR with human specific primers for the single copy gene human beta globin [24, 21]. The amount of human DNA at 9 days, 60 days, and 120 days was 0.57%, 0.48% and 0.37%, respectively (Figure 2G). Given the low number of animals, quantitative data only provides rough estimates. Moreover, due to the well-established polyploidy in hepatocytes, the given PCR method is expected to underestimate the actual proportion of engrafted FH-hTERT, which is in line with our phenotypic observations. In any case, the comparable levels in five different animals at various time points provide proof of principle for stable engraftment of FH-hTERT in uPA/SCID mice. Notably, at all time points, FH-hTERT retained their original morphology and expressed human albumin (Figure 2H–2I).

### Formation of tumors from transplanted RGB-marked cells

**Figure 3:**
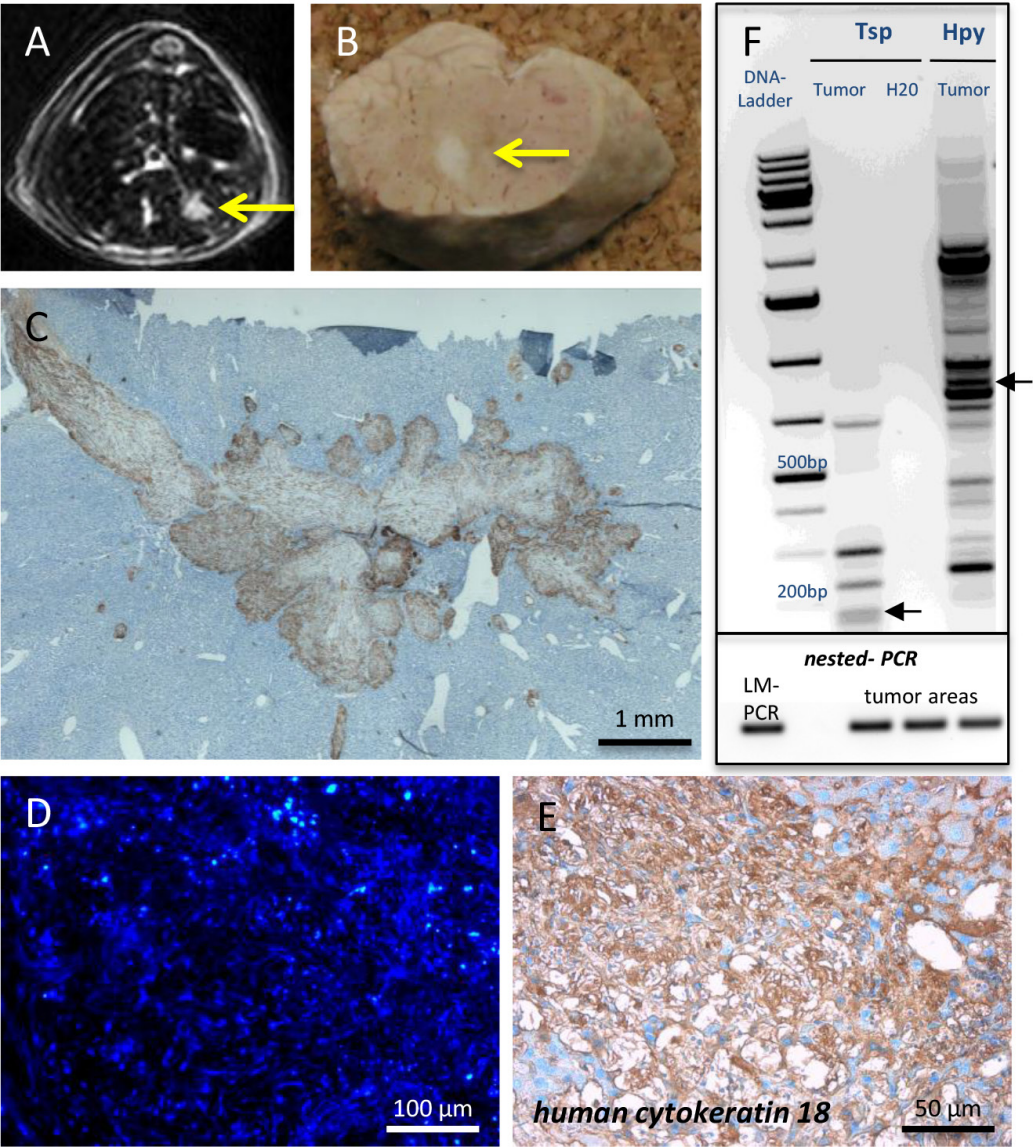
Tumor formation by transplanted FH-hTERT **(A-B)** 120 days after transplantation, MRI screening revealed tumor development in one mouse transplanted with RGB-marked FH-hTERT, which was macroscopically visible upon necropsy. **(C)** Immunohistochemistry for GFP and stitching of multiple microscopic pictures of the tumor visualized growth characteristics including invasion of surrounding mouse liver tissue as well as infiltration of blood vessels. **(D)** All cells of the tumor exhibited the same blue color, indicating monoclonal origin from a single transformed FH-hTERT. **(E)** Human origin of the tumor was confirmed by immunohistochemistry for human-specific cytokeratin 18. **(F)** Upper plot: LM-PCR of tumor DNA using the enzymes Tsp509I (Tsp) and HpyCH4IV (Hpy) followed by DNA sequencing (not shown) identified one lentiviral insertion site in the human genome (chromosome 5q/ arrow). Lower plot: Presence of this specific vector insertion in different areas of the tumor was confirmed by nested-PCR, proving clonal origin of the tumor from a single FH-hTERT.

Despite telomerase reactivation, FH-hTERT were not considered to be tumorigenic [25], but they can be transformed after insertional mutagenesis with gamma-retroviral vectors *in vitro* [11]. To date, orthotopic tumor formation in the liver by FH-hTERT has not been demonstrated. However, since liver tumor development resulting from lentiviral-vector mediated insertional mutagenesis was previously reported in other models [10], we performed Magnetic resonance imaging (MRI) in seven transplanted mice to screen for potential tumors, derived from RGB-marked FH-hTERT. In one mouse, MRI detected a liver tumor (diameter 4 mm), which subsequently was also visible macroscopically in the resected organ (Figure 3A–3B). Histopathological examination revealed characteristics of malignancy, including infiltration of surrounding liver tissue by the tumor as well as infiltration of blood vessels (Figure 3C). RGB marking showed that the FH-hTERT-derived tumor was characterized by homogenous blue coloring indicating its monoclonal origin and the presence of at least one LeGO-Cer vector insertion (Figure 3D). Immunohistochemistry for GFP (reactive also with Venus and Cerulean) as well as staining for human-specific CK18 verified that the tumor resulted from transplanted FH-hTERT (Figure 3E).

### Verification of tumor monoclonality

Individual integration patterns of RGB vectors in each transduced cell lead to specific homogenous coloring of clonally derived tissues, including tumors [14, 15]. To verify clonal origin of liver tumors arising from *per se* non-tumorigenic FH-hTERT in our model, we performed additional molecular analyses. As expected, LM-PCR identified only one lentiviral-vector integration site; here on chromosome 5q (Figure 3F, upper plot). To investigate whether the same specific integration site was also present in other parts of the same tumor, DNA was isolated from several tumor areas by microscopic dissection. Based on LM-PCR results, insertion-site specific primers were designed for amplification of the vector-genome junction by nested-PCR. Indeed, all tested tumor samples harbored the specific insertion site originally identified by LM-PCR, proving that the tumor was of clonal origin (Figure 3F, lower plot). In contrast, controls consisting of DNA isolated from other liver lobes of the same mouse containing transplanted RGB-marked FH-hTERT clusters but no tumor were all negative for this insertion (data not shown).

### Malignant transformation of FH-hTERT after insertional mutagenesis

Although a causal role of insertional mutagenesis was likely in the observed FH-hTERT-derived tumor, we did not identify genes or genetic elements potentially related to malignant transformation in proximity of the lentiviral integration site (200-kb window). However, since long-range effects of strong viral promotors – as used in LeGO vectors for RGB marking – were described [26, 27], this does not ultimately exclude an impact of the insertion on carcinogenesis.

**Figure 4:**
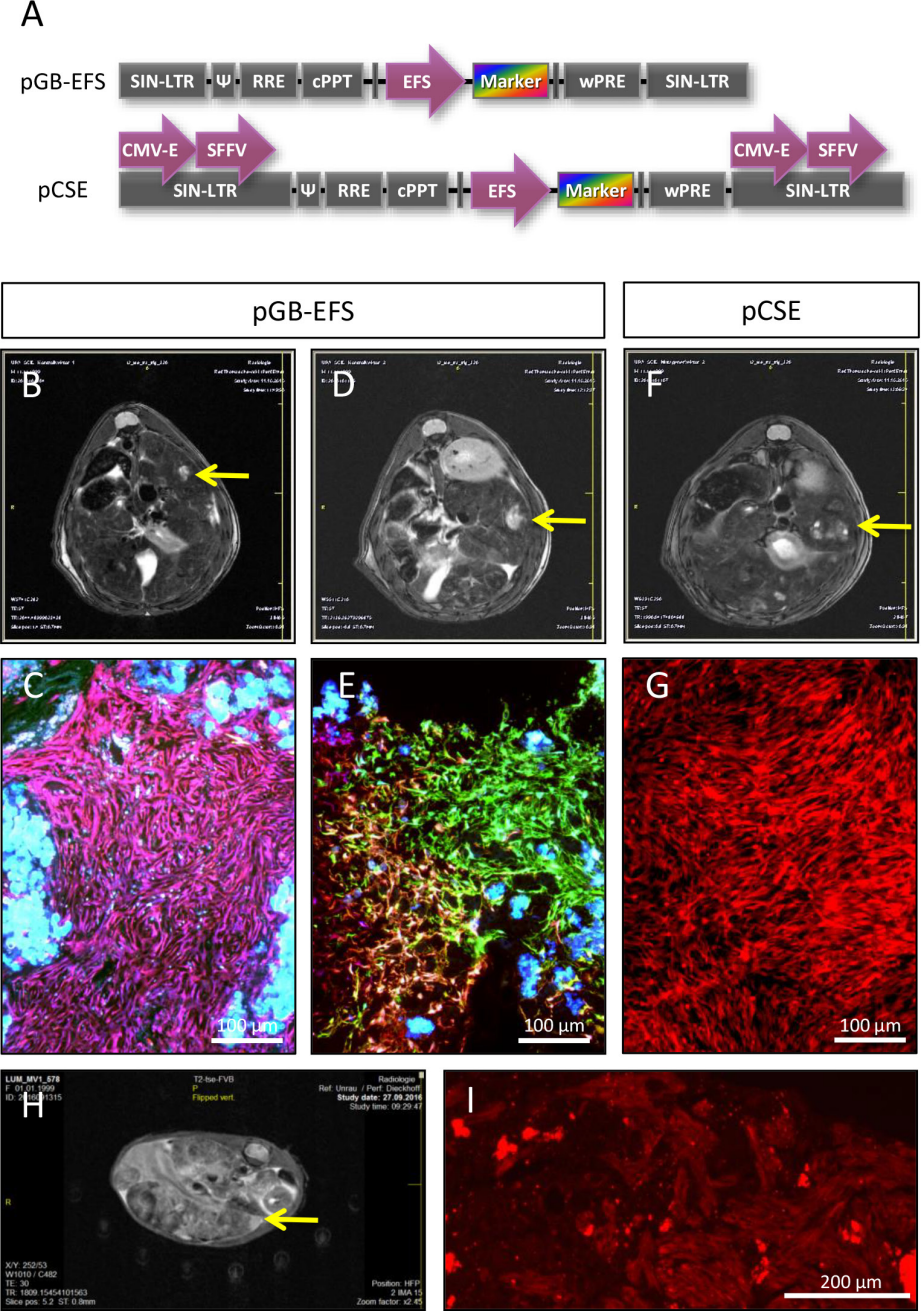
Formation of FH-hTERT derived liver tumors by additional lentiviral vectors **(A)** Design of pGB-EFS and pCSE lentiviral vectors for transduction of FH-hTERT differs in numbers of enhancers and promotors for potential transactivation of surrounding genes. Both vectors also include RGB fluorescent proteins. **(B-C)** 120 days after transplantation of pGB-EFS transduced FH-hTERT, a monoclonal liver tumor developed, as shown by MRI and fluorescence microscopy of liver sections (homogenously pink colored cells within the tumor). **(D-E)** A second tumor derived from pGB-EFS transduced FH-hTERT resulted from oligoclonal proliferation (six different colors were present within the tumor). **(F-G)** One mouse transplanted with pCSE transduced FH-hTERT developed a monoclonal (red) tumor. **(H-I)** Serial transplantation of red FH-hTERT derived tumor cells led to large liver tumors in three out of four hemizygous uPA/SCID mice 5 weeks after transplantation. (H) A representative MRT image five weeks after transplantation. (I) Cryosections were analyzed using the red channel of the fluorescence microscope. 10-fold magnification, scale bar 200 μm.

To further study potential mechanisms of FH-hTERT transformation in transplanted uPA/SCID mice, we cloned two additional lentiviral vectors with combinations of internal promotors and enhancers that potentially increase mutagenicity (Figure 4A). In mice transplanted with FH-hTERT transduced with lentiviral vectors with one internal promotor (EFS), MRI revealed tumor development in two out of three animals after 4 months. In mice transplanted with FH-hTERT transduced with lentiviral vectors carrying each one CMV enhancer and one SFFV promoter in both LTRs and an internal EFS promoter to drive marker expression, one out of four mice developed a tumor. In four control mice that received non-transduced FH-hTERT, no tumor development was detected.

Two of the three observed tumors were monoclonal according to RGB marking (Figure 4B–4C (pink) and Figure 4F–4G (red)), whereas the remaining one consisted of 6 different colors, indicating that this tumor was polyclonal arising from at least six different FH-hTERT clones (Figure 4D–4E). All tumors fulfilled histological criteria of malignancy, including infiltration of healthy liver tissue (Figure 4, and data not shown).

Since the red solid tumor penetrated the liver capsule we were able to isolate tumor cells by dissection of the superficial tumor. Resulting cells were re-cultured and a homogenously red tumor cell line evolved. Serial transplantation of these cells into new uPA/SCID mice resulted in fast development of multiple tumors in recipient mice as observed by MRI already 3 weeks post-transplant indicating further malignant progression of FH-hTERT-derived tumor cells (Figure 4H). Secondary tumors were homogenously red tumors proving their clonal origin (Figure 4I).

### Overexpression of MCF2 and MITF in FH-hTERT derived tumors

**Figure 5:**
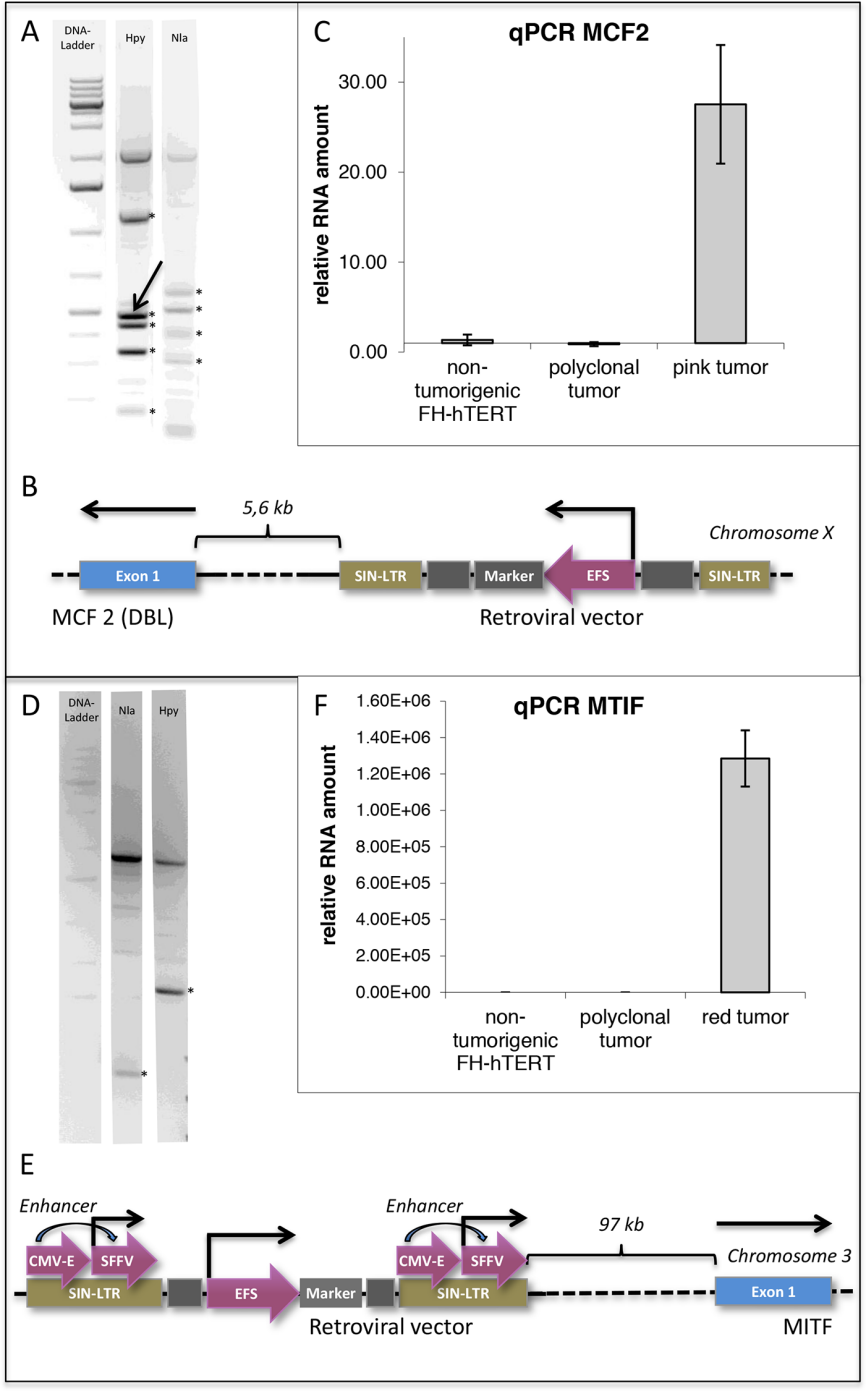
Detection of potential oncogenes by insertion-site analysis of monoclonal FH-hTERT derived liver tumors **(A)** LM-PCR of the pink liver tumor using two different enzymes identified five (Hpy) and four (Nla) lentiviral insertion sites in the genome of FH-hTERT (^*^). Since three of the revealed insertion sites were found with both enyzmes, a total of six independent insertion sites was retrieved. **(B)** One lentiviral insertion site was in proximity to the proto-oncogene MCF2 (DBL) (arrow). **(C)** qPCR confirmed 28-fold upregulation of MCF2 compared to original FH-hTERT and to the 6-colored polyclonal tumor. **(D-E)** In the red tumor only one lentiviral insertion site was detected (^*^), which was localized in proximity to transcription factor MITF. **(F)** Compared to original FH-hTERT and the polyclonal tumor, MITF was upregulated 1.29×10^6^-fold.

Liver-tumor formation in mice transplanted with lentiviral vector-transduced FH-hTERT, but not in control mice that received non-transduced FH-hTERT, indicates a probable role of insertional mutagenesis during malignant cell transformation. LM-PCR of the two monoclonal tumors was performed, and a 200-kb window around the identified insertion site was analyzed for flanking genes. In the pink monoclonal tumor (caused by the EFS vector) we identified an integration site in immediate proximity to the proto-oncogene MCF2/DBL (Figure 5A–5B). MCF2/DBL encodes for a guanine nucleotide exchange factor, and its aberrant expression has been described in human malignancies, albeit to date not in HCC [28–30]. To address the impact of the insertion on MCF2/DBL expression, we performed quantitative RT-PCR and detected a 28-fold increase in MCF2/DBL mRNA levels in the respective FH-hTERT-derived tumor, but not in primary FH-hTERT or in non-malignant regenerative FH-hTERT nodules in transplanted mouse livers (Figure 5C). These findings indicate upregulation of MCF2/DBL resulting from vector insertion. In the red tumor (caused by the CSE vector), we identified lentiviral integration in proximity of microphthalmia-associated transcription factor (MITF), a member of a family of transcription factors associated with melanoma development (Figure 5D–5E) [31–33]. Quantitative RT-PCR on tumor cells revealed massively elevated levels of MITF mRNA as compared to (i) primary FH-hTERT cells, (ii) non-malignant FH-hTERT regeneration clusters, but also (iii) the observed polyclonal tumor. Tremendous overexpression of MITF was also observed in dissected, isolated and re-cultured cells of the respective liver tumor (Figure 5F). In contrast to the FH-hTERT derived monoclonal tumors, no distinct DNA band could be visualized by LM-PCR in the polyclonal tumor, in agreement with the presence of multiple different integration sites.

### Overexpression of MCF2 and MITF in HCC cell lines and human HCC

Based on the positive results of MCF2/DBL and MITF upregulation in FH-hTERT-derived tumors in transplanted mice, we finally addressed a potential role of overexpression of both genes in human hepatocarcinogenesis. First we assessed potential dysregulation of the two genes in human HCC cell lines exploiting CellMinerHCC, a microarray-based expression database for HCC cell lines established by Teufel and colleagues [34]. For MCF2/DBL, only limited data for eight HCC cell lines is available not indicating transcriptional dysregulation. In contrast, MITF was found to be moderately upregulated in at least 13 of the 18 tested HCC lines, but not downregulated in any of them (Supplementary Figure 1). This finding is intriguing, since similar levels of upregulation were found in the database for established HCC proto-oncogenes such as H-RAS and SOX12. On the contrary, two genes (BRAF, FIGN) that were identified in a recent study using a different IM approach [10] were not found to be upregulated in the CellMinerHCC database (Supplementary Figure 1) [all data reference: http://medicalgenomics.org/cellminerhcc; accessed Oct. 19, 2017].

Next, expression levels of MCF2/DBL and MITF were measured by qPCR in 24 human HCC samples as well as in the respective surrounding non-tumorous tissue of the same surgically resected patient livers (Supplementary Table 1). Upregulation of MCF2/DBL was detected in five out of 24 HCCs (21%), whereas the gene was unchanged or downregulated in the remaining cases. Underlying diseases in the upregulated cases were chronic HBV (n=2), hepatitis C (n=1), and non-alcoholic steatohepatitis (NASH) (n=1). Two of the patients with chronic hepatitis (one HBV, one hepatitis C) had developed liver cirrhosis. MITF was upregulated in eight cases (33%), and etiologies were: chronic HBV (n=2), hepatitis C (n=3), and NASH (n=1); two hepatitis C and one HBV patients had established liver cirrhosis.

## DISCUSSION

Retroviral including lentiviral vectors stably integrate as proviruses into the host cell genome. As a consequence, vector integration can influence expression of cellular genes, which in the worst case may result in malignant transformation as seen in mouse models [7, 8, 35], but also in clinical gene therapy [36, 37]. Based thereon, insertional mutagenesis with replication-defective retroviral vectors has become a powerful tool to identify genes involved in various processes of malignant transformation and tumor outgrowth in different tissues [38]. At the same time, IM-mediated malignant transformation represents a rare event [39], and its probability depends on the respective cell type. This has been particularly elaborated in the hematopoietic system, where stem cells are much more prone to transformation than terminally differentiated T lymphocytes [40], even though the latter also do have a high proliferation capacity.

There has been strong evidence that IM also plays a role in HCC. For example, stable integration of subgenomic HBV sequences in hepatocytes affecting regulatory mechanisms has been suggested to be involved in HCC development [3]. Recent work highlighted the role of L1 retrotransposition in HCC [41]. Moreover, there have been reports demonstrating that adeno-associated virus (AAV) vector and even AAV wild-type insertions can cause malignant transformation of hepatocytes [42, 43], although data for the latter is controversial [44]. Based on those studies, IM with lentiviral vectors was successfully tested in various (tumor-prone) mouse models using fetal or newborn mice [10, 45]. In contrast, lentiviral transduction of adult hepatocytes did not result in tumor formation even after three serial transplantations [46].

In the present study we aimed at using human-derived cells (FH-hTERT) in an orthotopic xenograft model, since species differences between murine and human cells have to be taken into account when such studies are used as preclinical models for human hepatocarcinogenesis. FH-hTERT cells immortalized by expression of the proto-oncogene hTERT have been previously shown to engraft and survive in the liver of immunodeficient mice [18]. At the same time, despite their high growth rate *in vitro* there was no definite proof for proliferative activity *in vivo*. It is reasonable to assume that only a limited number of additional “hits”, potentially just one, are necessary for malignant transformation of these cells. Therefore, FH-hTERT represent an interesting target for IM-mediated hepatocarcinogenesis. We here combined IM with a chronic liver failure/regeneration model, i.e. the uPA/SCID mouse [19, 20]. The permanent loss of endogenous murine hepatocytes in this model in conjunction with the consecutive inflammatory response should induce proliferative stress, which is relevant for development of HCC as a strongly inflammation-associated malignancy.

For the first time we demonstrate stable engraftment and liver repopulation by FH-hTERT cells. Although FH-hTERT did not adopt typical hepatocyte-like morphology eight weeks after transplantation, remaining in their original rather spindle-cell shaped form, cells produced albumin as a characteristic hepatocyte-specific protein. Quantitative-PCR data on genomic DNA showed that repopulation rates were at least in the range of 0.5% of total liver mass. Although mice homozygous for the uPA transgene would potentially allow higher repopulation rates [20, 47], we here used hemizygous mice, in which recombined endogenous murine hepatocytes that lost uPA transgene expression can also participate in liver regeneration [48]. We reasoned that FH-hTERT-mediated regeneration would not be fast enough to save homozygous mice that die soon after birth due to liver failure. Therefore, since endogenous regeneration interferes with FH-hTERT proliferation, the obtained repopulation rates of ≥0.5% can be considered successful. The administration of the alkaloid MCT may have added to this success, since we did not observe reconstitution of uPA/SCID livers by FH-hTERT in the absence of MCT in previous pilot experiments (Lütgehetmann, unpublished). MCT disrupts the sinusoidal liver endothelium facilitating incorporation of transplanted cells, such as hepatocytes and liver sinusoidal endothelial cells into the hepatic parenchyma [22, 23]. The feasibility of liver repopulation with FH-hTERT allowed us to assess the impact of IM for hepatocarcinogenesis. We propose that our model is a valid tool that offers the potential to study other aspects of HCC development in a humanized orthotopic model in future experiments.

For labeling of FH-hTERT we applied multi-color RGB marking that enables clonality assessment of liver tumors in murine xenograft models [14]. The three lentiviral vectors used for RGB marking expressing red, green and blue fluorescent proteins integrate randomly but stable into the genome of the target cells generating mixed colors inherited to all daughter cells, thereby allowing analysis of clonal expansion. Use of RGB marking enabled visualization of repopulation kinetics of FH-hTERT in murine livers. Indeed, based on specific color labeling we initially observed polyclonal engraftment clusters 9d after transplantation. Out of these clusters, only some cells proliferated, which resulted in oligoclonal regeneration areas in the liver.

In our first experimental setting of long-term follow-up of RGB-marked FH-hTERT-transplanted mice, we detected one liver tumor in one out of seven mice observed long-term. In the other six mice, repopulated areas consisted of mono- or oligoclonal FH-hTERT without signs of malignant transformation. Although tumor formation was a singular event, this was the first demonstration of malignancy development after FH-hTERT transplantation [18]. Molecular cloning of the insertion site, followed by insertion-specific PCR proved that the tumor was monoclonal as suggested by RGB marking. We did not identify potential proto-oncogenes in proximity of the vector insertion, but several long non-coding RNAs have been described in the respective region of the long arm of chromosome 5. Moreover, long-range effects of retroviral insertion sites over hundreds of kb were reported [26]. Finally, it cannot be completely excluded that transformed cells contained further insertions not detectable by the used LM-PCR method [49].

To confirm IM-mediated malignant transformation of FH-hTERT, we repeated the transplantation experiments. However, in order to increase the likelihood of insertional mutagenesis, we introduced two modifications: (i) We transduced more cells per group to increase the probability of a malignant “hit” and (ii) we used novel RGB vectors containing several enhancers and promotors aiming to force IM. In addition we studied two further experimental groups of transplanted mice; receiving either FH-hTERT transduced with lentiviral RGB vectors containing an EFS promoter or FH-hTERT without lentiviral transduction (“control”). Engraftment of transplanted FH-hTERT was similar to the first experiment. None of the control animals developed a tumor. In contrast, three out of seven mice that received lentiviral-transduced FH-hTERT developed tumors, where it unexpectedly appeared to be irrelevant which of the above-mentioned vectors was used. While this finding confirms the initial proof-of-concept result, numbers of transplanted mice were too low to imply statistically sound conclusions regarding differences between the two vector types.

Two of the three observed tumors were monoclonal, whereas the third one was polyclonal consisting of at least six different clones. In addition, we were able to sustain one of the tumors *ex vivo* and prove its malignant phenotype and monoclonality by serial transplantation.

For both monoclonal tumors, we identified insertion sites of the integrated LeGO vectors. Notably, in both cases proto-oncogenes were located in close proximity to vector insertions. The first, MCF2/DBL is a guanine exchange factor (GEF) that activates GTP-binding proteins such as Cdc42, Rac, and Rho. This gene has been involved in carcinogenesis and progression of a variety of malignant diseases [28–30]. The other, MITF plays an important role during malignant transformation of melanocytes [31–33]. Both genes were strongly upregulated in FH-hTERT-derived tumors.

Since none of the two genes had been previously associated with liver cancer, we assessed their transcription levels in human HCC cell lines and HCC samples. For cell-line analysis we made use of the CellMinerHCC [34], a comprehensive microarray-based expression database for HCC cell lines. According to that data, MCF2 shows essentially no dysregulation in HCC cell lines. In contrast, we found a clear tendency towards moderate transcriptional upregulation of MITF in HCC cell lines. Notably, levels of upregulation were comparable to those of established, HCC-related proto-oncogenes such as H-RAS or SOX12. Moreover, in primary HCC we found upregulation of MCF2 in 21% and MITF in 33%. Even though the dataset is too small to draw any definite conclusions, it is intriguing to note that chronic infections with HBV and HCV seemed to be overrepresented in the etiology of HCC associated with increased expression of MCF2 and MITF (Supplementary Table 1).

Altogether, our data indicates that MCF2/DBL and MITF could play a role during malignant transformation of hepatocytes, although their actual impact needs to be evaluated in larger studies.

In conclusion, our findings demonstrate that human-derived FH-hTERT can be successfully used for repopulation of mouse livers, and that RGB marking of these cells is applicable to study their malignant transformation and progression on a clonal level. We have provided proof-of-principle that the joint use of RGB marking and insertional mutagenesis opens new possibilities to induce liver tumors and to study underlying molecular mechanisms. We therefore propose that this approach will be useful to explore molecular cancerogenesis as well as clonality of tumor growth in various malignancies.

## MATERIALS AND METHODS

### Cells and RGB marking

Cell characteristics and culture conditions of FH-hTERT were described previously [18]. Generation of lentiviral vectors, vector plasmids and lentiviral-vector mediated RGB marking of FH-hTERT by LeGO-C2 (Addgene No. 27339), LeGO-V2 (Addgene No. 27340) and LeGO-Cer2 (Addgene No. 27338) were described in detail [14, 15]. 5×10^4^ or 1×10^6^ cells were transduced at a multiplicity of infection (MOI) of 1 for each vector. In order to eliminate untransduced FH-hTERT, fluorescence-positive cells were sorted by fluorescence-activated cell sorting (FACS) on a FACS AriaIIIu (BD Biosciences, Heidelberg, Germany), using three different BP Filters (670 LP, 530/30, 510/50 nm). Flow-cytometry analysis was performed using FACS Canto II and BD FACS Diva Software 6.1.3 (both BD Biosciences).

### Cell transplantation

Alb-uPA transgenic SCID (uPA/SCID) mice were housed and maintained under pathogen-free conditions. To permeabilize the sinusoidal endothelium, mice received 200 mg/kg monocrotaline 16-24 hours before cell transplantation [22, 23]. Mice were anesthetized with inhaled isoflurane. The spleen was isolated by laparotomy and 1×10^6^ FH-hTERT in 40 μl DMEM+GlutaMAX with 2% FBS were injected into splenic pulp (Figure 1). Animal experiments were approved by local authorities and performed in accordance with relevant guidelines and regulations for animal welfare.

### Tissue analyses

Fixation of excised livers with paraformaldehyde (PFA), tissue dehydration in sucrose, snap freezing, and cryotome sectioning were essentially performed as described [14]. To visualize RGB-marked cells, we performed fluorescence microscopy of liver sections and generated overlays for the three basic colors red, green and blue detected in three different channels as described [14]. Immunohistochemistry was carried out using the following primary antibodies for 1h incubation: anti-green fluorescent protein (GFP) (1:300; Invitrogen, Karlsruhe, Germany) [50], anti-human cytokeratin 18 (1:100; Antibodies-Online, Aachen, Germany) [51], and anti-human albumin (1:100; DAKO, Hamburg, Germany). Visualization was performed with DAKO *REAL EnVision Kit* (DAKO) according to the manufacturer’s instruction. PFA-fixed cryosections were used for molecular analyses of lentiviral integration sites. Quantitative PCR for human beta globin gene was performed as described [52].

### Molecular analyses of clonality and gene expression

Isolation of DNA and RNA from PFA-fixed liver-cryosections was done following the manufacturer’s instructions (RNA/DNA isolation kit; Qiagen, Hilden, Germany). For detection of lentiviral insertion sites in the genome of FH-hTERT, we made use of ligation-mediated PCR (LM-PCR) as previously described [53, 54] using the restriction enzymes Tsp509I (Tsp), HpyCH4IV (Hpy) and NlaIII (Nla). Based on obtained sequence information we designed insertion-site specific primers to verify presence of specific clones in different areas of the same liver tissue by *nested*-PCR. The following primers were used: *PCR*: AGCTTGCCTTGAGTGCTTCA; TGCAGTTTTCTGGCTCTCCT, *nested-PCR*: AGTAGTGTGTGCCCGTCTGT; CAGTCTCTATGCAGTGGCTTT.

Expression levels of targeted genes were measured using the *SYBR Green* based real-time RT-PCR Kit and gene-specific *QuantiTect* primer assays (Qiagen) according to the manufacturer’s instructions: For expression of MCF2/DBL proto-oncogene in transplanted mouse livers and patient samples we applied human *Hs_MCF2_1_SG QuantiTect Primer Assay (QT00051429*) amplifying exon 20/21 expressed in all existing splice variants [55]. For expression of MITF transcription factor we used human *Hs_MITF_1_SG QuantiTect Primer Assay (QT00037737;* Qiagen), amplifying exons 3/4/5 expressed in all existing splice variants, which were characterized before [56]. Human GAPDH was used as reference gene (*Hs_GAPDH_1_SG QuantiTect Primer Assay (QT00079247),* Qiagen).

### Magnetic resonance imaging (MRI)

For detection of liver tumor development, MRI measurements of xenografted mice were carried out 4 months after cell transplantation and 3 to 5 weeks after retransplantation, respectively. Mice were imaged with a clinical 3.0 Tesla magnetic resonance scanner and a commercially available small-animal receiver coil as previously described [57], except for anesthesia, which was performed with inhaled isoflurane. In some experiments, a small-animal 7T MRI system was used.

### Gene expression analyses in human liver tissue

mRNA was isolated from 24 human HCC samples and the respective surrounding non-tumorous liver tissue. Patients gave their written informed consent and the local ethical committee of “Ärztekammer Hamburg” approved the use of resected tissue for research purpose (approval number PV3578). Quantitative RT-PCR for MCF2/DBL and MITF expression was performed as described above. Patient data is summarized in Supplementary Table 1.

## SUPPLEMENTARY MATERIALS FIGURE, TABLE AND REFERENCES



## Abbreviations

CMV: Cytomegalovirus
EFS: EF-1 (elongation factor 1) Alpha Short
FACS: Fluorescence-activated cell sorting
FH-hTERT: Human fetal hepatocytes expressing the human telomerase reverse transcriptase
HBV: Hepatitis B virus
HCC: Hepatocellular carcinoma
IM: Insertional mutagenesis
LeGO vectors: Lentiviral gene-ontology vectors
LM-PCR: Ligation-mediated PCR
MCF2: MCF.2 cell line derived transforming sequence (also named DBL)
MCT: Monocrotaline
MITF: Microphthalmia-associated transcription factor
MRI: Magnetic resonance imaging
RGB marking: Red-green-blue marking
SFFV: Spleen focus-forming virus
uPA/SCID: Urokinase-type plasminogen activator / severe combined immunodeficiency.
