# Supplementary material for: Multi-color RGB marking enables clonality assessment of liver tumors in a murine xenograft model
Source: Oncotarget. 2017 Dec 14;8(70):115582–95. doi: 10.18632/oncotarget.23312 (PMC5777795; doi:10.18632/oncotarget.23312)
Supplement: Supplementary file 1 [file oncotarget-08-115582-s001.pdf]

Multi-color RGB marking enables clonality assessment of liver tumors in a murine xenograft model

SUPPLEMENTARY MATERIALS

REFERENCES

1. Staib F, Krupp M, Maass T, Itzel T, Weinmann A, Lee JS, Schmidt B, Muller M, Thorgeirsson SS, Galle PR, Teufel A. CellMinerHCC: a microarray-based expression database for hepatocellular carcinoma cell lines. Liver Int. 2014; 34: 621-31. https://doi.org/10.1111/liv.12292.

2. Ranzani M, Cesana D, Bartholomae CC, Sanvito F, Pala M, Benedicenti F, Gallina P, Sergi LS, Merella S, Bulfone A, Doglioni C, von Kalle C, Kim YJ, et al. Lentiviral vector-based insertional mutagenesis identifies genes associated with liver cancer. Nat Methods. 2013; 10: 155-61. https://doi.org/10.1038/nmeth.2331.

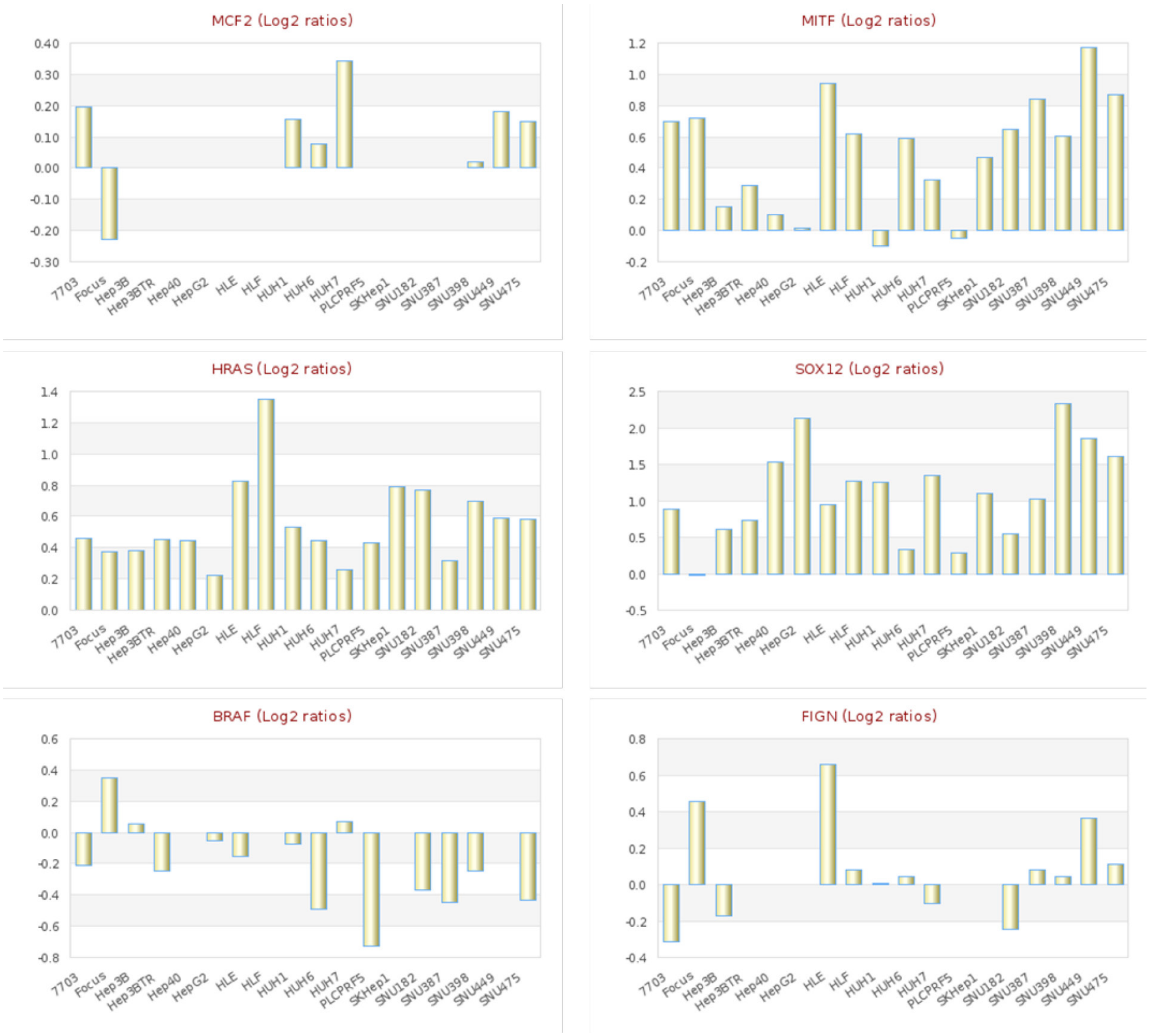

Supplementary Figure 1: Expression of MCF2/DBL and MITF in HCC cell lines. To assess expression of MCF2/DBL and MITF in HCC cell lines we made use of CellMinerHCC [1], a microarray-based expression database for hepatocellular carcinoma cell lines (http://medicalgenomics.org/cellminerhcc). Graphs were generated using the software implemented on the website [data obtained and processed Oct. 19, 2017]. Whereas no indication of transcriptional dysregulation was seen for MCF2, MITF was found to be moderately (>0.2) upregulated in 13 of the 18 tested HCC lines. Notably, similar levels of upregulation could be seen in the database for established proto-oncogenes such as H-RAS and SOX12. On the contrary, none of two contained HCC-causing genes (BRAF, FIGN) identified by insertional mutagenesis in a recent study [2] were found to be upregulated in the CellMinerHCC database.

Supplementary Table 1: Clinical characteristics of patients investigated in this study

	MCF2/DBL		MITF	
	**Downregulated/ unchanged**	**Upregulated**	**Downregulated/ unchanged**	**Upregulated**
**all (n=24)**	(n=19)	(n=5)	(n=16)	(n=8)
**Sex (male)**	84,2% (16)	100% (5)	93,8% (15)	75,0% (6)
**Age (mean)**	67,8	60,6	66,3	66,4
**Tumor stage**				
T1	31,6% (6)	60,0% (3)	37,5% (6)	37,5% (3)
T2	36,8% (7)	40,0% (2)	37,5% (6)	37,5% (3)
T3	31,6% (6)	0,0% (0)	25,0% (4)	25,0% (2)
**Grading**				
G1	15,8% (3)	60,0% (3)	25,0% (4)	25,0% (2)
G2	84,2% (16)	40,0% (2)	75,0% (12)	75,0% (6)
**Etiology/risk factors**				
NASH	15,8% (3)	20,0% (1)	18,8% (3)	12,5% (1)
ASH	10,5% (2)	0,0% (0)	12,5% (2)	0% (0)
HBV	15,8% (4)	40,0% (2)	25% (4)	25,0% (2)
HCV	21,1% (4)	20,0% (1)	12,5% (2)	37,5% (3)
**Liver cirrhosis**	36,8% (7)	40,0% (2)	37,5% (6)	37,5% (3)
**cryptogenic cirrhosis**	5,3% (1)	0,0% (0)	6,3% (1)	0% (0)
**no liver disease**	26,3% (5)	20,0% (1)	25,0% (4)	25,0% (2)

Abbreviations: NASH, non-alcoholic fatty liver disease; ASH, alcoholic liver disease; HBV, hepatitis B virus infection; HCV, hepatitis C virus infection.
